# Oxidative Stress in Animals: A Systematic Analysis from Signaling Pathways to Biological Effects

**DOI:** 10.3390/life16091516

**Published:** 2026-09-11

**Authors:** Le Chang, Chao Liu, Guangping Huang

**Affiliations:** Jiangxi Provincial Key Laboratory of Conservation Biology, College of Forestry, Jiangxi Agricultural University, Nanchang 330045, China; changle050704@163.com (L.C.); 15717968045@163.com (C.L.)

**Keywords:** oxidative stress, ROS/RNS, redox homeostasis, signaling pathway cross-talk, animal

## Abstract

Oxidative stress is caused by the imbalance between the generation of free reactive oxygen species (ROS)/reactive nitrogen species (RNS) and the antioxidant defense systems, which participate in animal growth, development, disease pathogenesis, and aging. This review summarizes endogenous and exogenous triggers of ROS/RNS overproduction, as well as model animals and cell models together with oxidative stress biomarkers and detection methods. We further dissect the three-layered redox regulatory network, focusing on crosstalk among four core pathways (Nrf2-ARE, NF-κB, MAPK, PI3K/Akt) that govern cell fate via oxidative eustress or distress. Excessive ROS/RNS trigger irreversible DNA damage and metabolic disorders, leading to inflammation and apoptosis. Finally, we prospect integrating non-model animals and multi-omics to advance oxidative stress research. This work provides a comprehensive theoretical framework for animal redox biology studies.

## Simple Summary

Oxidative stress arises from disrupted redox balance triggered by internal metabolism and external environmental factors, which causes damage to biological macromolecules and cellular structures. This review systematically summarizes the generation and inducing factors of ROS/RNS, common research models and detection techniques for oxidative stress, and the underlying molecular regulatory networks and biological effects. Furthermore, it prospects the applications of multi-omics technologies and non-model animals in future research.

## 1. Introduction

Early studies regarded ROS/RNS only as toxic molecules that induce cellular damages; however, with the deepening research, it has been recognized that ROS/RNS play important physiological roles in signal transduction and cell fate determination [1]. Under physiological concentrations, ROS/RNS serve as essential signaling molecules that regulate cell proliferation, immune responses, and metabolic activities; when ROS/RNS accumulate abnormally, however, intracellular redox homeostasis is disrupted, leading to irreversible oxidative damage to biological macromolecules [2].

Over long-term evolution, animals have evolved sophisticated multilayered regulatory networks to sense and regulate redox perturbations, including redox-sensitive sensing molecules and four canonical signaling pathways: nuclear factor erythroid 2-related factor 2-antioxidant response element (Nrf2-ARE), nuclear factor-κB (NF-κB), mitogen-activated protein kinase (MAPK), and phosphoinositide 3-kinase/protein kinase B (PI3K/Akt) signaling pathways [3]. These pathways do not operate independently; complex antagonistic, synergistic and feedback crosstalk among them jointly determines the ultimate cell fate.

But most current studies still focus on individual signaling pathways, lacking an integration of the entire oxidative stress cascade from molecular sensing to phenotypic output. In particular, the crosstalk among the core signaling pathways of oxidative stress has not been fully elucidated. To fill this gap, this review integrates the full chain of events from ROS/RNS generation and the oxidative stress molecular regulatory network to macromolecular damage, and constructs a complete research framework for animal oxidative stress. Our aim is to delineate the intact molecular cascade from redox signal perception and core pathway interactions to cascading damage to biological macromolecules, thereby providing a theoretical foundation for further exploration of oxidative stress mechanisms and translational insights for biomedicine practice.

Literature Search and Evidence Synthesis: This review on animal oxidative stress was built upon a structured multi-database literature search executed in PubMed/MEDLINE, Web of Science, Scopus and Google Scholar, supplemented by manual targeted retrieval of core animal physiology and redox biology journal archives, as well as supplementary web screening for recently released multi-omics and non-model animal research papers. The search window spanned from database inception to 10 August 2026. Combined search terms contained “oxidative stress”, “ROS”, “RNS”, “redox homeostasis”, “Nrf2-ARE”, “NF-κB”, “MAPK”, “PI3K/Akt”, “oxidative eustress”, “oxidative distress”, “oxidative stress biomarkers”, “animal model”, “non-model animal”, “multi-omics”, “lipid peroxidation”, “DNA oxidative damage”, “protein carbonylation”, and their derivative combined phrases. Reference lists of all retrieved original research articles, systematic reviews and thematic meta-analyses were manually back-screened to capture overlooked pioneering publications and cross-disciplinary comparative studies.

## 2. Generation and Triggers of ROS/RNS

### 2.1. Generation Pathways of ROS/RNS

ROS are highly reactive oxygen derivatives, including free radicals such as superoxide anion (O_2_•^−^), hydroxyl radical (•OH), hydrogen peroxide (H_2_O_2_) [4]. According to the source, ROS can be divided into endogenous and exogenous ROS [5]. Endogenous ROS is the product of intracellular aerobic metabolism, and the generation pathways can be divided into mitochondrial and non-mitochondrial routes [6]. About 90% of endogenous ROS are generated during the oxidative phosphorylation in the mitochondria [7]. Meanwhile, non-mitochondrial ROS generation is mainly mediated by cytosolic nicotinamide adenine dinucleotide phosphate (NADPH) oxidase complexes, which is the most representative enzyme system generating ROS independent of mitochondria [8]. Whereas exogenous ROS is mainly produced by exogenous substances, such as ultraviolet radiation (UV), industrial chemicals, and pollution [9].

Similar to ROS, RNS are highly reactive nitrogen derivatives, typified by nitric oxide (NO) and peroxynitrite anion (ONOO^−^) [10]. Based on the source, RNS can also be divided into endogenous and exogenous RNS. Endogenous RNS is predominantly synthesized by the catalysis of nitric oxide synthase (NOS), which exists in three major isoforms: neuronal NOS (nNOS), endothelial NOS (eNOS), and inducible NOS (iNOS) [11]. Exogenous RNS derives from environmental nitrogen oxides, nitrogen-containing drugs and atmospheric pollutants [9].

### 2.2. Main Triggers of ROS/RNS

Oxidative stress is not a random event but results from the synergistic effects of exogenous stress and endogenous metabolic abnormalities. These two classes of triggers collectively constitute the initiation system for oxidative stress in animals [12]. They significantly differ in duration of action, target sites, and core damage characteristics. The specific characteristics of various triggers are shown in Table 1.

Specifically, exogenous triggers originate from the external environment, including heavy metals [13], UV [14], chemical drugs [15], nutritional imbalances [16], temperature stress [17,18]. These factors can disrupt redox balance by damaging mitochondrial structure, inhibiting antioxidant enzyme activity, or activating intracellular signaling pathways, leading to oxidative stress.

In contrast, endogenous oxidative stress originates from aberrant intracellular metabolism dominated by disordered mitochondrial function. Excessive electron leakage of the mitochondrial electron transport chain (ETC) produces massive ROS, which is the dominant source of intrinsic oxidative insults [19]. Meanwhile, inflammatory cascades activated by intracellular damage signals upregulate NADPH oxidase and iNOS, further boosting the generation of ROS and RNS. Furthermore, aging [20], epigenetic abnormalities [21] and mutations in antioxidant genes [22] are also major triggers of endogenous ROS bursts.

## 3. Research Models and Methods

Suitable research models and detection methods are important for identifying the triggers of ROS/RNS and elucidating how ROS/RNS mediate the initiation of oxidative stress in organisms.

### 3.1. Research Models

Animal models and in vitro cell models are the primary experimental models applied in oxidative stress research. Animal models reflect whole-organism responses, while cell models can precisely control variables. These two categories complement each other and jointly drive progress in oxidative stress research.

Animal models have prominent advantages including physiological similarity, genetic editability and high experimental reproducibility [23]. Commonly used animal models include: mice, rats, zebrafish, *Caenorhabditis elegans*. Different animal species exhibit unique strengths; the optimal animal model varies depending on the research techniques and research objectives adopted in experiments. Due to mature genetic manipulation systems and high genomic homology, mice are the preferred experimental animals for gene-editing research on oxidative stress [24]. Rats are widely utilized in induced oxidative stress models for redox biomarker evaluation, as they provide abundant tissue samples and stable stress phenotypes [25]. With optically transparent larvae, high fecundity, a short developmental cycle, high capacity for genetic editing, and high genomic homology, zebrafish represent the optimal model for in vivo visualization of ROS and RNS [26,27]. *C. elegans* is highly suitable for rapid screening of aging-related oxidative stress, because of its short lifespan, conserved aging regulatory pathways and easy large-scale cultivation [28].

In vitro cell models facilitate well-controlled single-variable manipulation and high-throughput screening for oxidative stress research. It can be divided into three categories: immortalized cell models, primary cell models and functionally specific cell models. For immortalized cell lines, they feature stable genetic backgrounds, unlimited proliferation capacity and relatively straightforward transfection and gene-editing operations. Primary cells retain intact physiological characteristics, and their cellular signaling pathways and redox homeostasis are consistent with those of in vivo tissues [29]. Functionally specific cell models are derived from specialized tissue types with unique biological functions. They can precisely recapitulate tissue-specific oxidative stress processes, facilitating research on organ-targeted redox injury and the corresponding protective mechanisms [30].

### 3.2. Categorization of Core Oxidative Stress Indicators and Detection Methods

Selecting appropriate detection methods according to research objectives is of great significance for the study of oxidative stress. Detection techniques for oxidative stress can be divided into two categories: direct detection and indirect detection. Direct detection focuses on the measurement of reactive species, namely the direct quantification of ROS/RNS; while indirect detection performs qualitative or quantitative analysis by evaluating antioxidants and oxidative damage. Notably, no single assay can assess the overall oxidative stress level in biological samples (cells, tissues, organs or whole organisms). Multiple approaches from different dimensions are required to accurately and comprehensively evaluate the redox status [31]. Detailed information on common oxidative stress biomarkers and their corresponding detection assays are summarized in Table 2.

## 4. Molecular Regulatory Network of Oxidative Stress

**Figure 1:**
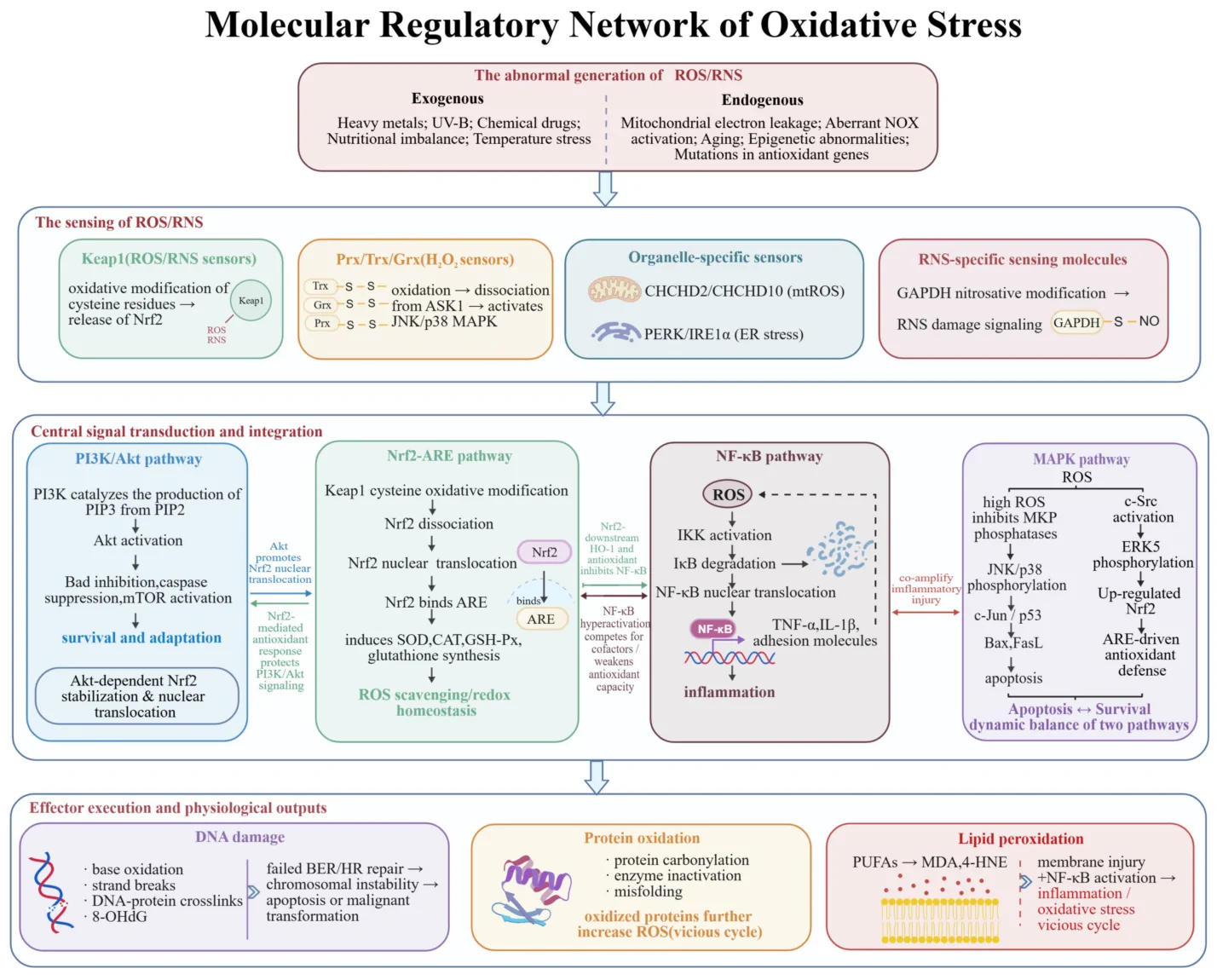
Molecular regulatory network of oxidative stress. Exogenous and endogenous stimuli trigger excessive generation of ROS/RNS. Multiple redox-sensing proteins detect redox perturbations and transduce signals to four core signaling cascades (Nrf2-ARE, NF-κB, MAPK, and PI3K/Akt). Complex crosstalk among these pathways determines cell fate. Persistent oxidative stress induces DNA damage, protein oxidation and lipid peroxidation, driving cellular dysfunction and pathological outputs.

Oxidative stress is not only the abnormal accumulation of ROS/RNS, but the consequence of the combined action of diverse intrinsic molecular regulatory networks within organisms [43]. The molecular regulatory network of oxidative stress possesses three core functions: signal sensing and discrimination, effector execution, and feedback regulation. It can be roughly divided into three tiers: upstream generation and sensing of ROS/RNS, central signal transduction and integration, as well as downstream effector execution and physiological output [44]. We constructed a schematic diagram in Figure 1 to illustrate the molecular regulatory network of oxidative stress.

### 4.1. The Generation and Sensing of ROS/RNS

Abnormal accumulation of ROS/RNS acts as the initiating factor triggering oxidative stress [1]. Once the redox balance is broken, intracellular redox-sensitive molecules can sense abnormal fluctuations in ROS/RNS levels [45]. These molecules often contain highly reactive cysteine thiol groups, which are susceptible to oxidative or nitrosative modifications induced by H_2_O_2_, lipid peroxides, ONOO^−^ and other substances [46]. And these modifications trigger conformational changes of proteins, thereby converting chemical oxidative signals into biological signals that can be transmitted within cells. The currently identified core sensors of ROS/RNS can be classified into four major categories:

The primary sensor of the cytoplasmic antioxidant pathway—Kelch-like ECH-associated protein 1 (Keap1): possesses a dual sensing capability to respond to modifications by both ROS and RNS. Under physiological conditions, Keap1 acts as the substrate adaptor for the Cullin 3 (Cul3)-Rbx1 E3 ubiquitin ligase complex; it binds to Nrf2 via a hinge-and-latch bimodal interaction, continuously targeting Nrf2 for ubiquitination and proteasomal degradation, thereby sequestering Nrf2 in the cytoplasm and abolishing its transcriptional activity [47]. Upon oxidative stress, key cysteine residues on Keap1 are oxidatively modified, which alters its protein conformation and releases Nrf2, thereby preventing its ubiquitin-mediated degradation. Consequently, Nrf2 accumulates abundantly in the cytoplasm and translocates into the nucleus to activate cellular antioxidant programs for the maintenance of redox homeostasis [48].

Hydrogen peroxide-specific sensing molecules: Mainly include peroxiredoxins (Prx family), thioredoxin (Trx), and glutaredoxin (Grx). These molecules exhibit ultrahigh sensitivity to intracellular H_2_O_2_ and function as both antioxidant scavengers and redox signal transducers via redox relay cascades. Under physiological conditions, reduced Trx directly binds to and inhibits Apoptosis signal-regulating kinase 1 (ASK1) via its thiol groups. Meanwhile, Prx and Grx act cooperatively to scavenge basal intracellular H_2_O_2_ and help maintain Trx in a reduced state, thereby keeping downstream MAPK pathways (JNK/p38) silent. In contrast, under oxidative stress, the thiol moieties of Prx, Trx and Grx undergo oxidative modification, Trx dissociates from ASK1 to activate ASK1-dependent MAPK cascades [49]. This pathway is involved in signal transduction related to oxidative damage, such as inflammatory responses and apoptosis [50].

Organelle-specific sensors: Are predominantly localized in the mitochondria and the endoplasmic reticulum (ER). In mitochondria, Coiled-coil-helix-coiled-coil-helix-domain-containing protein2/Coiled-coil-helix-coiled-coil-helix-domain-containing protein 10 (CHCHD2/CHCHD 10) are mitochondrial intermembrane space proteins that preserve mitochondrial cristae architecture, membrane potential and respiratory chain integrity. Their functional deficiency disrupts mitochondrial homeostasis and leads to secondary accumulation of mitochondrial ROS (mtROS), serving as indirect monitors of mitochondrial redox dysfunction [51]. In the ER, PERK, IRE1α and Activating transcription factor 6 (ATF6) constitute the three core unfolded protein response (UPR) branches. Oxidative stress disrupts ER calcium homeostasis and triggers misfolding protein aggregation, which dissociates the ER chaperone GRP78 from PERK/IRE1α/ATF6 to activate UPR signaling. Mild ER stress initiates adaptive damage repair, whereas persistent excessive stress switches the cascade toward inflammatory activation and apoptotic execution [52].

RNS-specific sensing molecules: RNS-dependent redox signal transduction relies on glyceraldehyde-3-phosphate dehydrogenase (GAPDH), a cysteine-containing protein sensitive to S-nitrosative modification. Excessive RNS mediates S-nitrosative modification on the catalytic cysteine residue of GAPDH; post-translational modification enables GAPDH to bind the E3 ubiquitin ligase Seven in absentia homolog 1 (Siah1), and the GAPDH-Siah1 complex translocates into the nucleus to transduce nitrosative stress signals and initiate damage-related apoptotic cascades [53].

Redox sensors with different functions cooperate and coordinate with each other to form an intact cellular redox perception network. This network achieves precise recognition, amplification and graded transduction of signals derived from abnormal ROS/RNS accumulation, and subsequently triggers a series of downstream biological processes.

### 4.2. Central Signal Transduction and Integration

After various sensors perceive the ROS/RNS, multiple intracellular signaling pathways transduce and integrate the sensed signals to generate regulatory outputs. Rather than functioning independently, these signaling pathways form a multilayered nonlinear crosstalk that collectively determines the ultimate cell fate. The four core signaling pathways involved in oxidative stress and their interaction mechanisms are described below.

Nrf2-ARE signaling pathway: The “key regulator of the antioxidant response” for cellular antioxidant defense—oxidative stress is regulated by the transcription factor Nrf2, which controls the expression of numerous antioxidant genes and is negatively regulated by Keap1. Under physiological conditions, Nrf2 binds tightly to Keap1 and undergoes continuous ubiquitination and degradation, maintaining low intracellular Nrf2 levels. Under oxidative stress, specific stress-sensing cysteine residues within Keap1 are oxidatively modified, inducing conformational changes in Keap1 and resulting in the dissociation of the Keap1–Nrf2 complex. In addition, the PI3K/Akt pathway promotes Nrf2 protein accumulation through an indirect mechanism. Activated Akt phosphorylates and suppresses GSK-3β activity. This event prevents GSK-3β-dependent phosphorylation of Nrf2 at the Neh6 degron domain and the following β-TrCP-mediated ubiquitination and degradation of Nrf2 [54]. Free Nrf2 translocates rapidly into the nucleus, heterodimerizes with Small musculoaponeurotic fibrosarcoma proteins (sMaf), and binds to antioxidant response elements (AREs) located in the promoter regions of target genes [55]. This transcriptional cascade upregulates antioxidant enzymes including SOD, CAT, GPX, heme oxygenase-1 (HO-1), as well as rate-limiting enzymes for glutathione biosynthesis (GCLC, GCLM), collectively boosting cellular ROS clearance capacity [56].

Notably, Nrf2 activation is a necessary but not sufficient condition for the restoration of redox homeostasis. Nrf2 activation alone only indicates that the cell has initiated antioxidant defense, it cannot be taken as evidence that redox homeostasis has been restored. To determine whether redox homeostasis has been restored, it is necessary to comprehensively evaluate multiple parameters, such as ROS levels, oxidative damage markers, and cellular physiological phenotypes. Meanwhile, the Nrf2-ARE signaling pathway exhibits strictly concentration- and time-dependent effects: transient moderate activation confers robust cellular protection against oxidative injury, while prolonged excessive Nrf2 activation contributes to pathological progression, including malignant tumor antioxidant drug resistance and tissue fibrosis [57].

NF-κB signaling pathway: The “central node linking oxidative stress and inflammation” linking oxidative stress and inflammation—NF-κB is a family of transcription factors that transactivate genes involved in various biological processes, including immune responses, inflammation, cell growth and survival [58]. Under physiological conditions, NF-κB dimers bind to IκB and are sequestered in the cytoplasm, losing transcriptional activity and maintaining intracellular homeostasis. Under oxidative stress, ROS-mediated IKK complex activation is primarily mediated by indirect upstream signal amplification. This process is driven by ROS-induced inactivation of cellular phosphatases and the subsequent activation of canonical upstream kinase cascades, including the TAK1 and PI3K/Akt signaling axes [59]. Once fully activated, the IKK complex undergoes autophosphorylation to acquire catalytic activity, mediating the phosphorylation and degradation of IκB proteins and liberating NF-κB dimers for nuclear translocation. Nuclear NF-κB binds to κB cis-acting elements of target genes and initiates the transcription of pro-inflammatory mediators including TNF-α and IL-1β, as well as cellular adhesion molecules, thereby triggering local inflammatory responses [60].

Notably, activated NF-κB transcriptionally upregulates NOX family subunits to boost endogenous ROS production, and recruited immune cells release massive extracellular ROS in inflammatory microenvironments. Dual ROS sources continuously sustain IKK activity, forming a vicious “oxidative stress-inflammation” positive feedback loop [61]. In contrast, extremely high ROS concentrations induce irreversible cysteine oxidation on IKKβ catalytic domains and suppress IKK kinase activity, acting as an intrinsic brake to avoid unrestrained inflammation [62].

MAPK signaling pathway: The “stress-responsive kinase module that modulates cell-fate decisions” mediated by oxidative stress—the MAPK pathway can be dually activated by ligand-bound receptors and various cellular stresses. Among them, four pathways (ERK, JNK, p38 and ERK5) can be phosphorylated and activated upon oxidative stress induction, whereas evidence for oxidative-stress-dependent activation of ERK3/4 is still scarce [63]. Under physiological resting conditions, the entire MAPK network is maintained in a dephosphorylated quiescent state via constitutive activity of MAPK phosphatases (MKPs); only ERK1/2 exhibits negligible basal activity, while JNK and p38 remain nearly fully silenced. Under oxidative stress, ROS reshapes the activity landscape of the four major MAPK axes via widespread cysteine oxidative modification. The JNK and p38 pathways are strongly activated while ERK activity is suppressed, and high concentrations of ROS inhibit MKP phosphatases, resulting in sustained phosphorylation of JNK and p38. The phosphorylated kinases translocate into the nucleus to further activate pro-apoptotic transcription factors such as c-Jun and p53, initiate the expression of apoptosis-related genes including Bax and FasL, and ultimately induce cellular apoptosis [64].

Unlike the pro-apoptotic effects described above, ERK5 is a functionally distinct member of the MAPK family that primarily mediates cytoprotection and antioxidant defense under oxidative stress. ERK5 is activated by phosphorylation via its upstream specific kinase MEK5, and this activation process is highly dependent on c-Src tyrosine kinase: H_2_O_2_ rapidly activates c-Src, which in turn activates ERK5, and c-Src deficiency completely blocks this pathway [65]. Activated ERK5 phosphorylates MEF2C and other MEF2 family transcription factors, promoting their binding to the Nrf2 promoter, thereby upregulating Nrf2 expression and initiating the transcription of ARE-driven antioxidant enzyme genes. Furthermore, experimental evidence indicates that knockdown or inhibition of ERK5 significantly exacerbates oxidative stress-induced cell death, thus confirming its protective role from a loss-of-function perspective. The ultimate fate of cells is determined by the dynamic balance between pro-apoptotic JNK/p38 signaling and ERK5-dominated protective responses [66].

PI3K/Akt signaling pathway: The “pro-survival pathway” under oxidative stress—the PI3K/Akt pathway engages in bidirectional regulatory crosstalk with oxidative stress. Acute activation mediates cytoprotection and cellular adaptation, whereas chronic overactivation drives pathological progression. Its ultimate function is determined by factors including the intensity and duration of oxidative stress as well as cell types [66]. Under physiological conditions, normal levels of ROS activate PI3K, which catalyzes the production of PIP3 from membrane phospholipids. PIP3 recruits Akt to the cell membrane and facilitates Akt phosphorylation and activation. Activated Akt maintains cell survival through multiple mechanisms: first, it phosphorylates the pro-apoptotic protein Bad. Phosphorylated Bad is sequestered in the cytosol via binding to 14-3-3 proteins, which relieves the inhibitory effect of Bad on anti-apoptotic Bcl-2/Bcl-XL and promotes cell survival [67]; second, it suppresses the activation of caspase family proteins to block apoptotic cascades [68]; third, it triggers the mTOR pathway to promote cellular metabolism and proliferation [69]. In addition, Akt promotes Nrf2 activity primarily via GSK-3β inhibition rather than direct phosphorylation, which activates the Nrf2 antioxidant pathway and induces anti-inflammatory negative regulators to negatively modulate NF-κB, thereby curbing excessive inflammatory activation, sustaining redox homeostasis [70]. Under oxidative stress, excessive ROS induces oxidative damage to the active sites of PI3K and Akt, thereby inhibiting pathway function. Meanwhile, mitochondrial apoptotic pathways are activated, ultimately resulting in cell death [71].

Within the regulatory network of oxidative stress, the four aforementioned pathways jointly assemble an elaborate signal integration platform via multiple feedback and synergistic mechanisms. First, there exists a primarily antagonistic interplay between Nrf2 and NF-κB. Activation of Nrf2 suppresses NF-κB-driven inflammatory responses by upregulating downstream effector molecules such as HO-1 [72]. Conversely, persistent NF-κB activation may attenuate the antioxidant capacity of Nrf2 either through competing for transcriptional cofactors [73]. The net outcome of this antagonistic contest determines whether cells undergo “antioxidant adaptation” or “inflammatory injury”. Meanwhile, the PI3K/Akt pathway exerts positive regulation on Nrf2. Activated Akt promotes Nrf2 activity, primarily via GSK-3β inhibition rather than direct Nrf2 phosphorylation, generating a synergistic “antioxidation-prosurvival” effect. After eliminating ROS, antioxidant enzymes downstream of Nrf2 indirectly shield the PI3K/Akt pathway from oxidative damage, thereby forming a positive feedback loop conducive to cell survival. In addition, the MAPK pathway achieves bidirectional modulation of p53 and Nrf2. p38 MAPK phosphorylates and activates p53 to strengthen its pro-apoptotic function; meanwhile, MAPK signaling can alter the activity and stability of Nrf2 via phosphorylation modification [74]. Such dual regulation renders MAPK a critical “rheostat” for cell fate determination: it leans toward Nrf2-mediated survival under mild stress, yet switches to p53-triggered apoptosis upon severe stress [75]. Furthermore, NF-κB and MAPK exert synergistic amplification effects during inflammation. The two pathways are frequently co-activated. ROS-stimulated JNK/p38 enhances the transcriptional activity of AP-1, whereas NF-κB upregulates pro-inflammatory cytokine expression. Jointly, they cooperatively promote inflammatory reactions at the transcriptional level and aggravate tissue injury. Collectively, the Nrf2-ARE, NF-κB, MAPK and PI3K/Akt pathways constitute a dynamic nonlinear decision-making network through antagonistic and synergistic crosstalk as well as positive and negative feedback loops. This integrated network dictates the ultimate fate of cells confronting oxidative stress: survival and adaptation, inflammatory damage, or apoptosis [2].

### 4.3. Downstream Effector Execution and Physiological Output

Excessive ROS/RNS cause persistent, irreversible oxidative damage to intracellular macromolecules [76]. Such damage lays the molecular foundation for oxidative stress to evolve from molecular lesions into cellular dysfunction. Various forms of damage reinforce each other and generate a cascade amplification loop, and their long-term accumulation leads to cellular dysfunction and apoptosis [77].

DNA damage: ROS/RNS can directly attack DNA bases and the deoxyribose backbone, causing base oxidation, strand breaks, and DNA-protein crosslinks. 8-hydroxy-2′-deoxyguanosine (8-OHdG) is a characteristic marker [78]. If not promptly repaired by base excision repair (BER) or homologous recombination (HR), this damage can lead to chromosomal breaks and rearrangements, ultimately inducing apoptosis or malignant transformation.

Protein oxidation: Protein carbonylation is a characteristic marker. ROS/RNS specifically attack amino acid residues to form carbonyl groups, disrupting spatial conformation, leading to loss of enzyme activity and misfolding [79]. Accumulation of oxidized proteins further promotes ROS generation, forming a vicious cycle of “protein damage—ROS exacerbation” [80,81,82].

Lipid peroxidation: This primarily occurs in the phospholipid bilayer of cell membranes as a chain-propagating reaction. Polyunsaturated fatty acids (PUFAs) are the main targets [83]. The end products, malondialdehyde (MDA) and 4-hydroxynonenal (4-HNE), are highly cytotoxic, activate inflammatory pathways such as NF-κB, and create a “lipid oxidation-inflammation-oxidative stress” vicious cycle [84].

## 5. Biological Effects Mediated by Oxidative Stress

Through a series of downstream biological processes, oxidative stress converts intracellular redox imbalance and biomacromolecular damage into pathophysiological alterations at the tissue, organ and individual levels. Importantly, the biological effects triggered by oxidative stress exhibit marked dose-dependence and a dual nature: moderate levels of reactive oxygen species (ROS) trigger oxidative eustress, supporting cell communication, metabolic regulation, immune defense and adaptive responses; however, when ROS levels exceed the cellular antioxidant capacity, oxidative modifications of lipids, proteins and nucleic acids are initiated, which drive pathological outcomes [2].

### 5.1. Oxidative Eustress

Oxidative eustress constitutes a sophisticated regulatory mechanism indispensable to animal life activities. Its essence lies in the reversible signal transduction function exerted by moderate levels of ROS/RNS within physiological concentration ranges [1]. At the cellular level, hydrogen peroxide, as the major representative of ROS, operates at nanomolar concentrations. It selectively oxidizes redox-sensitive cysteine residues on target proteins to form reversible redox switches, thereby precisely governing pivotal signaling cascades including NF-κB, MAPK, PI3K/Akt and Nrf2, and participating in fundamental biological processes such as cell proliferation, differentiation, metabolism adaptation and immune response [85]. At the tissue and organismal level, oxidative eustress modulates a wide array of beneficial physiological outcomes. For instance, mitochondrial superoxide inhibits prolyl hydroxylases under hypoxic conditions to stabilize hypoxia-inducible factors (HIF), triggering angiogenesis and metabolic remodeling [86]. Therefore, oxidative eustress essentially relies on the dynamic cycle of continuous ROS generation and elimination to maintain high cellular sensitivity to oxidative stimuli, enabling fine-tuned regulation of bodily life processes [1].

### 5.2. Oxidative Distress

Oxidative distress refers to a pathological state in animals where ROS/RNS production outpaces the scavenging capacity of antioxidant systems, resulting in irreversible damage to biomolecules. Unlike oxidative eustress, ROS indiscriminately attack DNA, proteins and lipids under oxidative distress, triggering persistent damage accumulation, disruption of cellular homeostasis and activation of pro-inflammatory pathways, ultimately leading to cellular dysfunction, apoptosis or malignant transformation [1].

Notably, oxidative eustress and oxidative distress are not mutually exclusive; dynamic interconversion exists between them, which is determined by ROS concentration, exposure duration and cell type. Within the picomolar to nanomolar range, ROS primarily execute signal transduction functions. Once concentrations exceed this threshold, oxidative eustress transitions into oxidative distress, accompanied by a series of oxidative lesions [87].

## 6. Future Perspectives

Research on oxidative stress has evolved from initial oxidative damage to a systematic understanding of the redox signaling network. With the continuous development of gene editing, multi-omics, single-cell sequencing, and other technologies, significant progress has been made in elucidating the molecular mechanisms of oxidative stress [53]. However, current research still faces many scientific questions and gaps. Traditional model organisms, constrained by their domestication backgrounds, struggle to reflect the natural adaptations [88]. Moreover, single-omics analyses fall short of delineating the complex, multidimensional interplay that defines the redox network in its entirety [89]. In contrast, non-model animals offer a unique window into both the evolutionary conservation and lineage-specific divergence of oxidative stress mechanisms [90]. And multi-omics integration enables the systematic consolidation of regulatory hubs across the gene-protein-metabolite layers, thereby furnishing a comprehensive molecular cartography of the redox landscape [91].

### 6.1. Innovative Construction of Non-Model Animals and Elucidation of Adaptive Mechanisms

At present, model animals such as mice and rats are extensively employed in fundamental research on oxidative stress. However, owing to multiple generations of artificial inbreeding and selective breeding, together with standardized laboratory husbandry, model animals differ substantially from non-model animals in physiological traits such as redox signaling pathways and oxidative damage repair efficiency. Non-model animals refer to species that are not established as standard laboratory organisms and therefore have not undergone extensive artificial inbreeding, selective breeding, or standardized husbandry. Consequently, they retain natural genetic diversity and remain exposed to natural environmental conditions, in contrast to the homogenized genetic backgrounds and controlled environments of model animals. To systematically and clearly compare these divergent profiles of oxidative stress response, we have compiled a detailed comparison table, which is presented as Table 3 in the main text.

As shown in the table, the regulatory mechanisms of oxidative stress feature both evolutionary conservation and pronounced interspecific heterogeneity. Utilizing non-model animals as research subjects can overcome the inherent limitations of conventional model organisms in terms of evolutionary background, natural selection pressure and genetic diversity. From the perspectives of species specificity and molecular mechanisms, such research offers novel insights into deciphering the regulatory network of oxidative stress.

### 6.2. Integrating Multi-Omics to Enhance the Precision of Oxidative Stress Network Regulation Studies

Oxidative stress is a complex, multi-level regulatory process involving multiple genes, proteins, and metabolites, characterized by high cellular heterogeneity and spatiotemporal specificity. Applying any single omics technique in isolation suffers from one-sided limitations. For instance, transcriptomics, will detect biomolecules of one type and thus can only capture changes in a small subset of the biological cascade [89]. As illustrated in studies on oxidative stress-triggered senescence in rat nucleus pulposus cells, the combination of transcriptomics, untargeted metabolomics and single-cell RNA sequencing identified a key metabolic bottleneck: glutamate depletion. Even with elevated transcription of synthases, antioxidant capacity remained compromised [102]. Taken together, multi-omics strategies consolidate data from multiple molecular levels to systematically characterize the intricate regulatory network of oxidative stress, providing a robust toolkit to further dissecting oxidative stress molecular mechanisms.

## Figures and Tables

**Table 1:** Classification and characteristic features of oxidative stress triggers.

Inducer Type	Time Course	Representative Examples	Main Targets	Core Damage Characteristics
Exogenous	Rapid (seconds to minutes)	Heavy metals, UV-B, chemical drugs,	Mitochondria structure, antioxidant enzymes, cell membrane	Direct inhibition of antioxidant enzyme activity; instantaneous ROS burst; acute oxidative injury
	Slow (days to months)	Nutritional imbalance, temperature stress	Signaling pathways, immune regulatory system	Activation of inflammatory pathways; formation of a chronic oxidative-inflammatory cycle
Endogenous	Rapid (minutes to hours)	Mitochondrial ETC electron leakage, aberrant NOX activation	Mitochondria, redox system	Excessive endogenous ROS/RNS production; rapid collapse of redox balance
	Slow (months to years)	Aging, epigenetic abnormalities, mutations in antioxidant genes	Antioxidant regulatory pathways, gene expression regions	Gradual decline of the antioxidant system; low-grade chronic oxidative stress

**Table 2:** Common biomarkers in oxidative stress and corresponding detection assays.

Classification	Marker	Description and Detection Methods	Reference Nos.
Direct detection markers	ROS	The most intuitive indicator of oxidative stress intensity. Captured by fluorescent dyes or radical trapping agents; its dynamic levels are positively correlated with oxidative injury severity.	[32]
Direct detection markers	RNS	Comprises NO, ONOO^−^ and other derivatives. Detected by fluorescent probes; its level is positively correlated with the extent of oxidative/nitrosative damage.	[33]
Indirect detection markers—Lipid peroxidation products	MDA	One of the typical end products of membrane lipid peroxidation, increased MDA levels mean aggravated oxidative stress. Tested via TBARS colorimetry or fluorometry.	[34,35]
Indirect detection markers—Lipid peroxidation products	4-HNE, 8-iso-PGF_2_α, LOOH	Supplementary lipid oxidation biomarkers, reflecting the degree of oxidative attack on cell membranes and lipoproteins. 4-HNE: ELISA/IHC/LC-MS; 8-iso-PGF_2_α: ELISA/GC-MS; LOOH: FOX/HPLC.	[36,37]
Indirect detection markers—Antioxidant enzymes	SOD	Catalyzes dismutation of superoxide anion radicals. Its activity and gene expression levels evaluate the radical scavenging ability of organisms. Assessed by activity assay kits (WST-1/NBT method) or qPCR/WB.	[34,38]
Indirect detection markers—Antioxidant enzymes	CAT	Breaks down hydrogen peroxide into water and oxygen; its activity reflects intracellular H_2_O_2_ clearance efficiency. Measured by UV spectrophotometry (monitoring H_2_O_2_ consumption at 240 nm) or activity assay kits.	[39]
Indirect detection markers—Antioxidant enzymes	GPX, POD, GST, GSH-PX, GR, TPX	Combined measurement of these enzymes enables comprehensive evaluation of total antioxidant capacity. Activities determined by substrate-specific spectrophotometric assays (e.g., GPX: NADPH oxidation; GST: CDNB conjugation).	[40]
Indirect detection markers—Non-enzymatic antioxidants	GSH	Core non-enzymatic antioxidant that directly scavenges free radicals; its content indicates the functional state of the non-enzymatic antioxidant system. Determined by DTNB colorimetric assay (GSH/GSSG ratio) or HPLC.	[34,38]
Indirect detection markers—Protein oxidative damage	Protein carbonyls	Classic marker of protein oxidative injury, measurable through colorimetric assays or ELISA.	[38]
Indirect detection markers—DNA oxidative damage	8-OHdG	Gold-standard biomarker for DNA oxidation; quantified by ELISA, comet assay or immunofluorescence staining.	[38,41]
Indirect detection markers—Mitochondrial function	MMP	Closely linked to redox homeostasis. Fluorescent probes are applied to monitor oxidative stress-induced mitochondrial dysfunction.	[42]

Malondialdehyde (MDA), Thiobarbituric Acid Reactive Substances (TBARS), 4-Hydroxynonenal (4-HNE), 8-isoprostaglandin F2α (8-iso-PGF_2_α), Lipid Hydroperoxides (LOOH), Enzyme-Linked Immunosorbent Assay (ELISA), Immunohistochemistry (IHC), Liquid Chromatography-Mass Spectrometry (LC-MS), Gas Chromatography-Mass Spectrometry (GC-MS), Ferrous Oxidation-Xylenol Orange assay (FOX), High Performance Liquid Chromatography (HPLC), Superoxide Dismutase (SOD), Water Soluble Tetrazolium salt-1 (WST-1), Nitro Blue Tetrazolium (NBT), Quantitative Real-Time Polymerase Chain Reaction (qPCR), Catalase (CAT), Reduced Glutathione (GAH), Oxidized Glutathione (GSSG), 5,5′-Dithio-bis-(2-nitrobenzoic acid) (DTNB), Glutathione Peroxidase (GPX), Peroxidase (POD), Glutathione S-transferase (GST), Glutathione Reductase (GR), Thioredoxin Peroxidase/Peroxiredoxin (TPX), 1-Chloro-2,4-dinitrobenzene (CDNB), Western blot (WB).

**Table 3:** Oxidative stress heterogeneity between model and non-model animals.

Comparison Dimension	Model Animals	Non-Model Animals	Reference Nos.
Basal antioxidant background level	Possess a low and inducible antioxidant baseline. Nrf2 is continuously degraded via the Keap1–Cul3 pathway under normal conditions	Exhibit constitutive upregulation of antioxidant genes in wild subterranean rodents. Naked mole-rats show sustained Nrf2 activity but lower GSH levels and higher oxidative damage	[47,92,93,94,95]
Nrf2-ARE pathway activation characteristics	Nrf2 activation is inducible and transient with a short half-life (<20 min); the pathway rapidly deactivates after stress cessation	Nrf2 activation is inducible and transient with a short half-life (<20 min); the pathway rapidly deactivates after stress cessation	[47,92,93]
ROS kinetic response under equivalent stress	Produce intense ROS bursts with high peaks and delayed clearance	Show reduced mitochondrial ROS production. Bats exhibit lower oxygen-coupled H_2_O_2_ release, potentially contributing to milder oxidative stress; systematic interspecies comparison data remain insufficient	[96,97]
Oxidative damage repair system efficiency	Have limited and saturable repair capacity. Deficiencies in core antioxidant genes cause oxidative hypersensitivity or embryonic lethality	Maintain high antioxidant expression but do not show superior damage clearance. Their stress tolerance may depend on enhanced proteostasis rather than rapid oxidative damage repair	[92,98,99,100,101]

## Data Availability

No new data were created or analyzed in this study. Data sharing is not applicable to this article.

## Abbreviations

The following abbreviations are used in this manuscript:

4-HNE	4-hydroxynonenal
8-OHdG	8-hydroxy-2′-deoxyguanosine
8-iso-PGF_2_α	8-isoprostaglandin F_2_α
ARE	Antioxidant-response element
ASK1	Apoptosis signal-regulating kinase 1
BER	Base-excision repair
CAT	Catalase
CDNB	1-Chloro-2,4-dinitrobenzene
CHAC1	Glutathione-specific gamma-glutamylcyclotransferase 1
CHCHD	Coiled-coil-helix-coiled-coil-helix-domain-containing protein
DTNB	5,5′-Dithio-bis-(2-nitrobenzoic acid)
ELISA	Enzyme-Linked Immunosorbent Assay
ETC	Electron transport chain
FOX	Ferrous Oxidation-Xylenol orange assay
GCLC	Glutamate-cysteine ligase catalytic subunit
GC-MS	Gas Chromatography-Mass Spectrometry
GPX	Glutathione peroxidase
GR	Glutathione reductase
Grx	Glutaredoxin
GSH	Glutathione
GSSG	Oxidized glutathione
GST	Glutathione S-transferase
HO-1	Heme oxygenase-1
HPLC	High performance liquid chromatography
HR	Homologous recombination
HIF	Hypoxia-inducible factors
IHC	Immunohistochemistry
IL	Interleukin
iNOS	Inducible nitric-oxide synthase
IRE1α	Inositol-requiring enzyme 1α
JNK	c-Jun N-terminal kinase
Keap1	Kelch-like ECH-associated protein 1
LC-MS	Liquid Chromatography-Mass Spectrometry
LOOH	Lipid hydroperoxides
MAPK	Mitogen-activated protein kinase
MDA	Malondialdehyde
MKP	Mitogen-activated protein kinase phosphatase
MMP	Mitochondrial membrane potential
mTOR	Mammalian target of rapamycin
mtROS	Mitochondrial reactive oxygen species
NADPH	Nicotinamide adenine dinucleotide phosphate
NBT	Nitro Blue Tetrazolium
NER	Nucleotide-excision repair
NF-κB	Nuclear factor-kappa-B
NLRP3	NOD-like receptor family pyrin-domain-containing 3
NOX	NADPH oxidase
NOS	Nitric oxide synthase
Nrf2	Nuclear factor-erythroid 2-related factor 2
PERK	Protein kinase RNA-like endoplasmic reticulum kinase
PHLDA2	Pleckstrin-homology-like domain family A member 2
PI3K	Phosphoinositide-3-kinase
PIP3	Phosphatidylinositol 3,4,5-trisphosphate
POD	Peroxidase
Prx	Peroxiredoxin
PUFA	Polyunsaturated fatty acid
qPCR	Quantitative Real-Time Polymerase Chain Reaction
RNS	Reactive nitrogen species
ROS	Reactive oxygen species
sMaf	Small musculoaponeurotic fibrosarcoma
SOD	Superoxide dismutase
TBARS	Thiobarbituric Acid Reactive Substances
TNF-α	Tumor necrosis factor-alpha
TPM3	Tropomyosin 3
TPX	Thioredoxin Peroxidase/Peroxiredoxin
Trx	Thioredoxin
TrxR	Thioredoxin reductase
UV	Ultraviolet radiation
WB	Western blot
WST-1	Water Soluble Tetrazolium salt-1
*C. elegans*	Caenorhabditis elegans
